# Mathematical Modeling of Triphasic Viral Dynamics in Patients with HBeAg-Positive Chronic Hepatitis B Showing Response to 24-Week Clevudine Therapy

**DOI:** 10.1371/journal.pone.0050377

**Published:** 2012-11-28

**Authors:** Hwi Young Kim, Hee-Dae Kwon, Tae Soo Jang, Jisun Lim, Hyo-Suk Lee

**Affiliations:** 1 Department of Internal Medicine and Liver Research Institute, Seoul National University College of Medicine, Seoul, Republic of Korea; 2 Department of Mathematics, Inha University, Incheon, Republic of Korea; 3 Division of Gastroenterology and Hepatology, Seoul National University Boramae Medical Center, Seoul, Republic of Korea; Osaka University Graduate School of Medicine, Japan

## Abstract

**Background:**

Modeling of short-term viral dynamics of hepatitis B with traditional biphasic model might be insufficient to explain long-term viral dynamics. The aim was to develop a novel method of mathematical modeling to shed light on the dissociation between early and long-term dynamics in previous studies.

**Methods:**

We investigated the viral decay pattern in 50 patients from the phase III clinical trial of 24-week clevudine therapy, who showed virological response and HBsAg decline. Immune effectors were added as a new compartment in the model equations. We determined some parameter values in the model using the non-linear least square minimization method.

**Results:**

Median baseline viral load was 8.526 Log_10_copies/mL, and on-treatment viral load decline was 5.683 Log_10_copies/mL. The median half-life of free virus was 24.89 hours. The median half-life of infected hepatocytes was 7.39 days. The viral decay patterns were visualized as triphasic curves with decreasing slopes over time: fastest decay in the first phase; slowest in the third phase; the second phase in between.

**Conclusions:**

In the present study, mathematical modeling of hepatitis B in patients with virological response and HBsAg decline during 24-week antiviral therapy showed triphasic viral dynamics with direct introduction of immune effectors as a new compartment, which was thought to reflect the reduction of clearance rate of infected cells over time. This modeling method seems more appropriate to describe long-term viral dynamics compared to the biphasic model, and needs further validation.

## Introduction

Chronic hepatitis B virus (HBV) infection is a serious global health problem, affecting approximately 350 million people worldwide. [1] Currently there are several approved therapeutic agents against HBV infection including interferon and nucleos(t)ide analogues (NUCs) such as lamivudine, adefovir, entecavir, telbivudine and tenofovir. However, current treatment for hepatitis B is not yet satisfactory in that these agents, especially NUCs, do not eradicate HBV, only to maintain sustained suppression of HBV replication and relapse is common even after HBV DNA being undetectable if treatment is discontinued. [2], [3] Recently, mathematical modeling of the dynamics of human immunodeficiency virus (HIV) and hepatitis C virus (HCV) infection during antiviral therapy has contributed to the development of effective treatment strategies by enabling the prediction of treatment response.[4]–[6] Hence, it is of particular importance to understand the viral dynamics of HBV infection more in detail.

Previous studies on HBV viral dynamics during antiviral treatment have provided valuable insights into the complex interaction between the host and the virus in patients with chronic hepatitis B (CHB).[7]–[15] Commonly employed procedures for modeling in these studies were based on the standard biphasic model developed by Nowak et al., which consists of rapid decay of free circulating virions followed by slower reduction of infected hepatocytes [7]. The biphasic model was largely based on the analysis of viral dynamics focused on the early part of treatment period (as short as 4 weeks) in the majority of these studies. Unlike HCV, however, early viral dynamics has been suggested to be insufficient to predict long-term dynamics because of the dissociation between early and long-term dynamics; in particular, long-term rather than early dynamics would be relevant in real clinical practice where antiviral treatment is considered for life-long in most patients. [16] Recently, an intriguing model proposed by Dahari et al. successfully described complex HBV DNA decay patterns under treatment by including hepatocyte proliferation and critical drug efficacy in their model equations. [17] However, challenging issues still remain in establishing a new modeling for the prediction of long-term treatment outcome. Direct incorporation of dynamic immune response into modeling was attempted in acute hepatitis B, but not in CHB. [18], [19] In addition, although subsequent phases following the second phase of early HBV dynamics have been identified, modeling beyond the first 2 phases has not been fit into the classic biphasic model. [20], [21].

We therefore aimed to describe HBV dynamics for longer period than previous studies by directly introducing the immune activity of cytolytic and noncytolytic HBV clearance in the mathematical modeling.

## Methods

### Mathematical Model for Viral Dynamics

We analyzed HBV viral dynamics using a modified model of viral infection originally suggested by Neumann et al. [6] Our model included four differential equations describing the change in the number of uninfected or target cells (“T”), infected cells (“I”), viral load (“V”), and immune effectors (“E”) with both cytolytic and noncytolytic activities. Target cells are susceptible to infection by circulating virions, and infected cells which contain covalently closed forms of HBV DNA (cccDNA) are able to produce free virions. Taking into consideration that chronic HBV infection lasts for decades, it has been assumed that the viral level stabilizes at a steady state at which the rate of viral production equals the rate of viral clearance. [22] Another assumption was that infected hepatocytes may generates infected daughter cells by mitosis and that both target and infected hepatocytes contribute to the liver cell turnover in proportional to their concentration. [23] Finally, the immune effectors are produced in response to the presence of infected cell presenting relevant antigens as well as preexisting immune effectors. Changes of serum alanine aminotransferase (ALT) level would reflect the robustness of cytolytic activity at the time of measurement which is harbored by the immune effector (“E”). This cytolytic activity was assumed to demonstrate negative feedback with reducing activity over time. [21] Although there is currently no established marker or measurement of noncytolytic immune activity during the process of chronic HBV infection, previous studies with animal models reported that the total HBV DNA amount in the liver can be decreased by noncytolytic immune activity, without a corresponding decrease in the number of infected hepatocytes. [24], [25] Quantified HBV surface antigen (HBsAg) levels might represent the amount of replicative HBV genome as well as HBV sequences integrated in the hepatocytes, providing relevant information on the state of residual HBV-infected hepatocytes during antiviral treatment. [26] Hence, we assumed that the noncytolytic activity of immune effectors (“E”) can be reflected, at least in part, by the changes of quantified serum HBsAg levels; serial HBsAg levels during treatment might represent change in the amount of HBV genome which is reduced indirectly due to the decrease of the infected cells by the cytolytic activity of “E” as well as reduced directly by the noncytolytic activity of “E” on the infected cells. Immune effector-induced clearance rate of the infected hepatocytes was designated as “α” in the equation of the infected hepatocytes (“I”). Immune effectors are also thought to exert noncytolytic activity, probably causing noncytolytic, cytokine-induced “curing” of infected cells. [24], [27] The extent of noncytolytic activity, although it cannot be exactly measured, was assumed to be related to the extent of cytolytic activity, and therefore noncytolytic activity was expressed using “α" with a calibration coefficient (“f”), in the equation of the target cells (“T”). The immune response assumed here is similar to the HIV model suggested by Bonhoeffer et al., with a Michaelis-Menten type saturation nonlinearity. [28] We also add a source term “S_E_” to characterize a non-zero steady state for immune effectors, “E”. [29] Thus, the dynamics during antiviral therapy were described as follows:
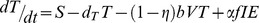


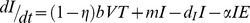


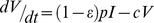


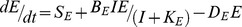



The model equations have several parameters which must be assigned for numerical simulation. We used literature values for some of the parameters, and they are listed in Table 1 with their description and assigned values. [20] It is an unresolved issue whether (and to what extent) de novo infection with HBV is blocked by treatment with NUCs (“η” in the above equation). This cannot be resolved by current data for viral dynamics, and therefore, a fixed value for η was assumed in previous studies, e.g., 1.0 (complete blocking) [8], [13], 0 (no blocking) [6], [30] or 0.5 (partial blocking). [10] A fixed value of 0.5 or 1.0 for η is known as a safer assumption for obtaining the rate of infected cell loss with less bias and a reasonable standard error. [31] Thus, we chose 0.5 for the value of η, i.e., partial blocking of de novo infection.

**Table 1:** The parameters and their assigned values.

Parameter	Value	Description
s	5×10^5^ cells/mL·d	production rate of target cells
d_T_	0.003	death rate of target cells
η	0.5	treatment efficacy of inhibiting *de novo* infection
b	4×10^(−10)^ mL/(copies·d)	*de novo* infection rate of target cells
f*		calibration coefficient of α for target cells
m	0.003	mitotic production rate of infected cells
d_I_	0.043	death rate of infectec cells
α*	7×10^(−4)^;	immune effector-induced clearance rate of infected cells
ε*		treatment efficacy of inhibiting viral production
p	6.24 d^−1^	viral production rate by infected cells
c	0.7	clearance rate of free virions
S_E_*		production rate of immune effectors
B_E_*		maximum birth rate for immune effectors
K_E_*		Michaelis-Menten type coefficient for immune effectors
D_E_*		death rate of immune effectors

NOTE. * These paremeters were solved numerically by fitting with the model equations in each patient.

Abbreviations: T, target cells; I, infected cells; E, immune effector with both cytolytic and noncytolytic activity.

The MATLAB®-ODE solver (MathWorks, Natick, MA, USA) was used to numerically solve our model equations. We performed the individual non-linear least-square minimization method, using the Levenberg-Marguardt algorithm in order to estimate the parameters which were not predetermined.

### Patients

The applicability of modeling was evaluated in a total of 182 hepatitis B e antigen (HBeAg)-positive CHB patients who were enrolled in the treatment group in a randomized, phase III clinical study of clevudine. Inclusion and exclusion criteria were previously described in detail. [32].

Among a total of 182 enrolled patients, 110 patients who achieved an arbitrarily defined “response” at the end of the 24-week treatment were assessed for the applicability of modeling; “response” was defined as decline of serum HBV DNA level and ALT to below 10,000 copies/mL and below ULN, respectively. These patients were distinctively divided into two groups according to the patterns of quantified HBsAg kinetics: one group showed decline of HBsAg titer during the 6-month treatment period (n = 62); the other group did not (n = 48). The latter group was discarded from analysis in the present study. Among the first group of 62 patients, 12 patients with fluctuating HBV DNA levels more than 1 Log_10_ copies/mL during treatment were excluded due to concerns about the accuracy of the mathematical processing. Hence, a total of 50 patients from this group were included in our final analysis. Patients were evaluated at baseline and monitored on days 8, 15, and 29 and every 4 weeks thereafter, during a total period of 24 weeks. The analysis was conducted using the quantified data of serum HBV DNA, HBsAg and ALT from the blood samples obtained at each time point.

The present study was approved by the institutional review board of Seoul National University Hospital. Written informed consents were obtained from all subjects. We conducted the study in accordance with the principles of the Declaration of Helsinki.

### Quantification of Serum HBV DNA, ALT and Serum HBsAg

Serum HBV DNA and ALT levels were obtained at each time of sampling used in the previous study. [32] Serum HBV DNA levels were measured at a central laboratory using a Digene Hybrid Capture II assay (Digene Corp., Gaithersburg, MD) with the lower limit of detection of 4700 copies/mL; the COBAS Amplicor PCR assay (Roche Molecular Systems, Branchburg, NJ, USA) with a lower limit of detection of 300 copies/mL was used to measure lower levels of HBV DNA when HBV DNA was undetectable by the Digene Hybrid Capture II assay.

Serum HBsAg titer was measured for the present study in serial serum samples from each patient which were stored at –70°C until the time of analysis. HBsAg was quantified using the Architect HBsAg assay (Abbott laboratories, Abbott Park, IL, USA; dynamic range, 0.05–250.0 IU/mL) after a 1∶100 dilution. Samples with HBsAg levels >250.0 IU/mL at 1∶100 dilution were retested at a final dilution of 1∶1000. Samples with HBsAg levels <0.005 IU/mL at 1∶100 dilution were retested undiluted.

### Statistical Analysis

Continuous variables were presented as either mean with standard deviation or median with interquartile range (IQR). Correlation between variables of baseline or follow-up and estimated parameters was calculated using Pearson’s correlation coefficients. Simple and multiple linear regression analyses were performed to identify independent determinants of parameters among variables of interest. A *P* value <0.05 was considered as statistically significant. All statistical tests were two-sided. Statistical analyses were performed using SPSS 17.0K (SPSS Inc., Chicago, IL, USA).

## Results

### Patient Characteristics at Baseline and During Treatment

The baseline characteristics of 50 enrolled patients were as follows: median age was 37 years; 32 patients were male (64%); median baseline ALT level was 124.5 IU/L; median baseline levels of HBV DNA and HBsAg were 8.526 Log10 copies/mL and 4.044 Log10 IU/mL, respectively. The HBV DNA data measured from baseline to the end of therapy were used to fit the model equations in the 50 selected patients with HBeAg-positive CHB. Table 2 demonstrates the baseline and on-treatment virologic characteristics of the enrolled patients (n = 50).

**Table 2:** Baseline and on-treatment characteristics of all patients (n = 50).

	median (range) or N(%)
Age (years)	37 (18–55)
Sex (male)	32 (64%)
Baseline HBV DNA (Log_10_ copies/mL)	8.526 (6.549–9.625)
Baseline ALT (IU/L)	124.5 (43–514)
Baseline HBsAg (Log_10_ IU/mL)	4.044 (3.031–4.805)
Changes in HBV DNA (Log_10_ copies/mL)	5.683 (4.004–7.148)
Changes in HBsAg (Log_10_ IU/mL)	0.304 (0.007–1.605)
Transition point between phase 1 and 2 (days)	7.83 (6.33–19.33)
Transition point between phase 2 and 3 (days)	33.17 (29.25–46.75)
Slope of phase 1 (Log_10_ copies/mL/day)	0.217 (0.093–0.252)
Slope of phase 2 (Log_10_ copies/mL/day)	0.073 (0.033–0.100)
Slope of phase 3 (Log_10_ copies/mL/day)	0.020 (0.019–0.022)

NOTE. All values are reported as median (range).

### Parameter Estimation

Estimated parameters are shown in Table 3. Fitting the data using the aforementioned numerical methods was successful in 50 enrolled patients. The antiviral efficacy was 94.7%. The median half-life of the free virus and of the infected cells was 24.89 hours (interquartile range (IQR), 22.68–27.10) and 7.39 days (IQR, 4.29–10.49), respectively.

**Table 3:** Estimated parameters by numerical methods.

	Median	(range)
α	7.54E-04	(6.25E-05–8.74E-04)
f	1.00E-01	(0–1.00E-01)
S_E_	9.33	(6.68–9.74)
B_E_	0.52	(0.47–0.53)
K_E_	4.07E+05	(9.25E+04–8.18E+05)
D_E_	0.50	(0.49–0.53)
ε	0.969	(0.966–0.982)
I_t 1/2_ (day)	7.39	(2.94–15.52)
V_t 1/2_ (hour)	24.89	(23.92–74.00)

NOTE. The meaning of each parameter is described in Table 1.

Estimates of the following parameters are reported as exponents: α, f, S_E_, B_E_, K_E_, D_E_.

Abbreviations: I_t 1/2_, half-life of infected hepatocytes; V_t 1/2_, half-life of free virus; SD, standard deviation.

### Viral Decline

**Figure 1:**
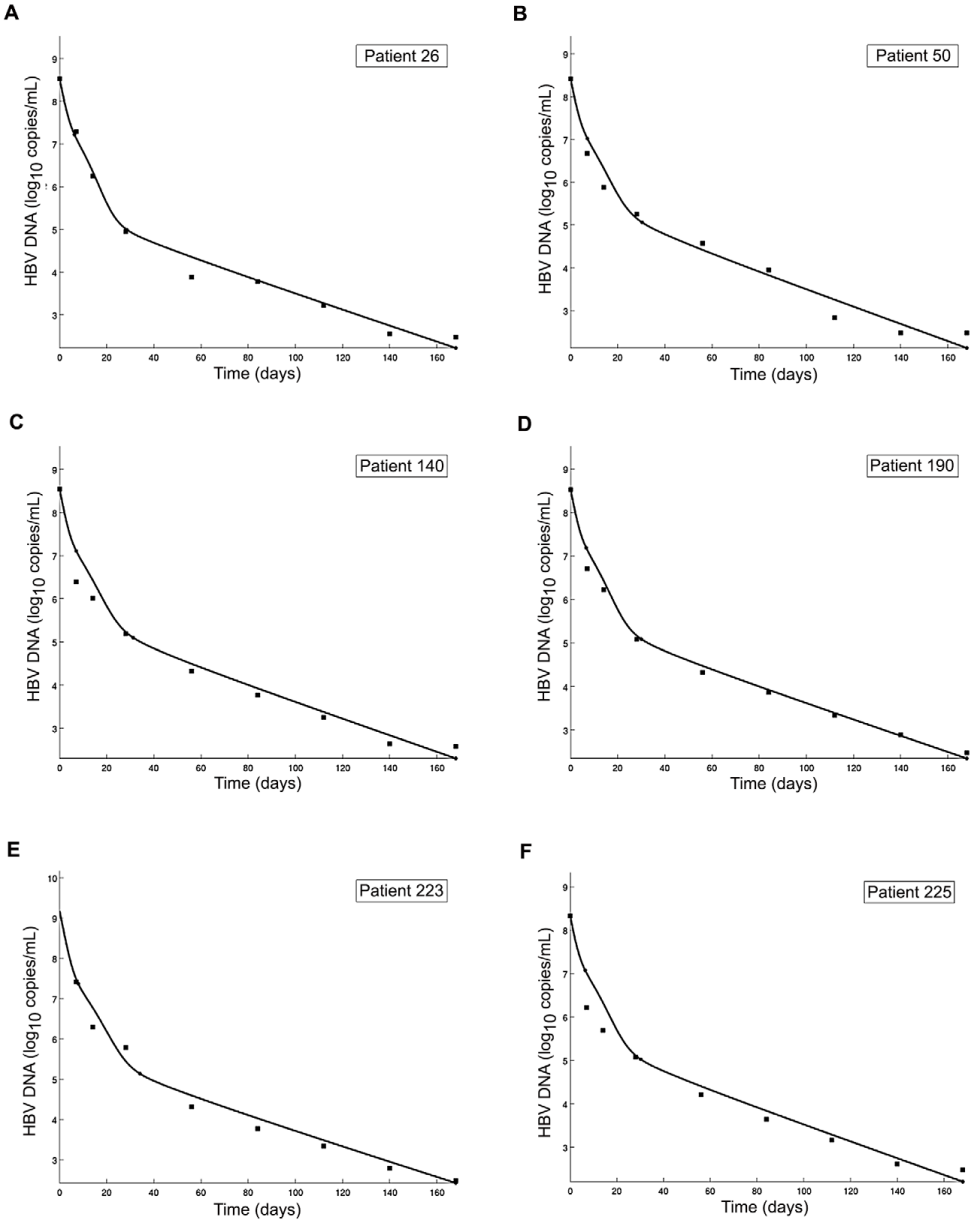
Examples of viral decay patterns with fitted curves during 24 weeks of clevudine therapy (Fig. 1-A–F). Squares represent quantified values of HBV DNA, and solid lines indicate fitted curves according to the modeling of viral dynamics in each patient. Baseline patient profiles (sex (M, male; F, female)/age/HBV DNA (Log10 copies/mL)/ALT (IU/L)/HBsAg (Log10 IU/mL)) are as follows: Patient 26, F/51/8.53/135/3.68; Patient 50, M/38/8.42/78/4.01; Patient 140, F/50/8.54/204/4.35; Patient 190, M/52/8.53/83/4.31; Patient 223, F/53/9.17/46/4.32; Patient 225, F/40/8.33/88/4.12.

Median viral load decline from the baseline levels measured at week 24 of the clevudine therapy was 5.683 Log_10_ copies/mL. In concordance with the original data, visualization of the calculated viral decay curves followed a triphasic pattern in these patients (Fig. 1 (A–F)). The first phase (phase 1) showed the fastest decay of the viral load; the third phase (phase 3) showed the slowest decay and the second phase (phase 2) was in between. The median estimate of the location of the transition point from phase 1 to phase 2 was 7.83 days; the median transition point between phase 2 and phase 3 was 33.17 days. The median values of slopes of phase 1, 2 and 3 were 0.217, 0.073 and 0.020 Log_10_ copies/mL/day, respectively.

### Correlation and Regression Analyses

**Figure 2:**
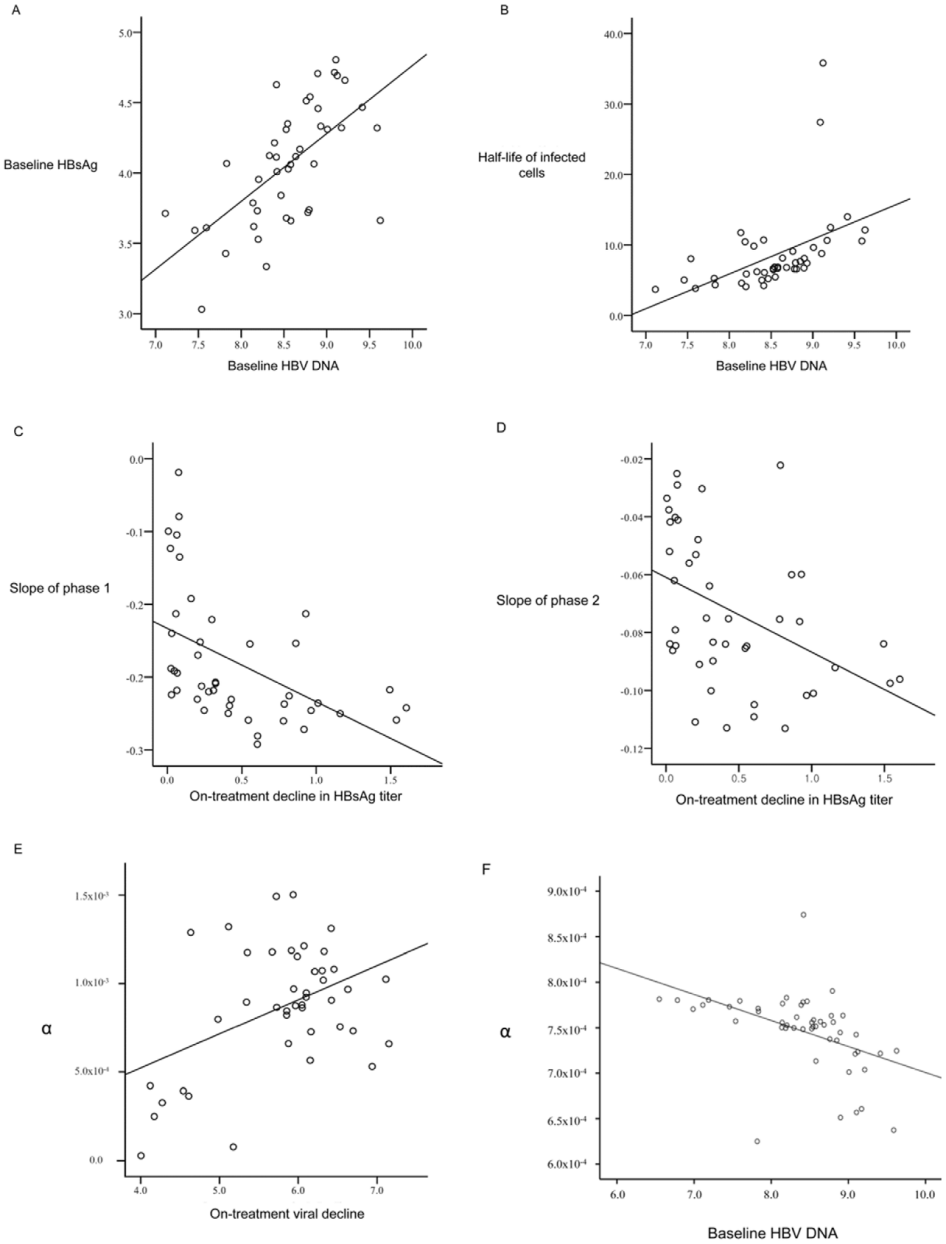
Linear regression analyses for the relationship between clinical characteristics and calculated parameters (Fig. 2-A–F). Baseline HBV DNA was significantly correlated with baseline HBsAg (2-A) and half-life of infected cells (2-B). Slopes of phase 1 and 2 showed correlation with on-treatment decrease in HBsAg titer (2-C,D). Calculated parameter “α”, or cytolytic activity-induced clearance rate of infected cells, was correlated with antiviral efficacy (2-E) and baseline HBV DNA (2-F). Abbreviations: HBV, hepatitis B virus; HBsAg, hepatitis B surface antigen.

Table 4 summarizes Pearson’s correlation coefficients for the relationship between clinical characteristics and calculated parameters. Fig. 2 demonstrates the results of linear regression analyses for these parameters. Baseline HBV DNA level was significantly correlated with baseline HBsAg titer (Pearson correlation coefficient (r) = 0.648, *P*<0.001; Fig. 2-A) and half-life of infected cells (r = 0.932, *P*<0.001; Fig. 2-B). Slopes of phase 1 (Fig. 2-C) and 2 (Fig. 2-D) showed correlation with on-treatment decrease in HBsAg titer (r = 0.501, *P*<0.001; r = 0.496, *P*<0.001). Among the calculated parameters in the model equations, parameter “α” (cytolytic activity-induced clearance rate of infected cells) was correlated with both antiviral efficacy (r = 0.337, *P* = 0.017; Fig. 2-E) and baseline HBV DNA (r = −0.466, *P* = 0.001; Fig. 2-F).

**Table 4:** Pearson’s correlation coefficients (r) for the relationship between clinical characteristics and calculated parameters.

Variables	Variables	R	*P*
Baseline HBV DNA	Baseline HBsAg titer	0.648	<0.001
Baseline HBV DNA	Half-life of infected cells	0.932	<0.001
On-treatment decrease in HBsAg titer	Slope of phase 1	0.501	<0.001
On-treatment decrease in HBsAg titer	Slope of phase 2	0.496	<0.001
α	Antiviral efficacy	0.337	0.017
α	Baseline HBV DNA	−0.466	0.001

Abbreviations: HBV, hepatitis B virus; α, immune effector-induced clearance rate of infected cells.

## Discussion

In the present study, we analyzed the viral dynamics during 24 weeks of clevudine therapy in 50 patients with HBeAg-positive CHB who also showed the decline of serum HBsAg titers during therapy. Triphasic dynamics were demonstrated by solving our mathematical models with direct incorporation of immune effectors with cytolytic and non-cytolytic activities in the equations in these patients. To the best of our knowledge, this study was the first attempt to mathematically solve the model equations including immune effectors in chronic hepatitis B to show triphasic dynamics.

Simulation of viral dynamics throughout long-term treatment requires additional assumptions for both the hepatocyte proliferation and the immune system activity. [33] We modified the model proposed by Neumann et al. [6] with introducing immune-mediated clearance of infected cells by cytolytic as well as noncytolytic activities of immune effector cells; these are reflected by serum ALT level and serum HBsAg titer, respectively. In our model equations, the activities of immune effectors were designated as directly cytolytic activity (parameter “α”) and noncytolytic activity (parameter “f”). According to our estimates, virion clearance rate (median half-life of 24.89 hours) was similar to those reported in previous studies conducted with less frequent blood sampling (Table 5); however, our virion clearance rate was longer than the values in three other studies involving shorter intervals between blood samples in the early phase of therapy. [11], [12], [15] The longer virion half-life in our results might be caused by the sparse schedule of early blood sampling which cannot reflect the delay in viral decay due to initial delay in action time attributed to pharmacokinetics of antiviral agent during the early period; therefore, the virion clearance rate is supposed to be underestimated with data from less frequent early sampling. [31] However, the virion clearance rate (phase 1 in our results) is a parameter of early treatment phase, and its relevance in the long-term kinetics might be of less importance compared to other parameters which are explored below.

**Table 5:** Comparison of viral dynamic parameters and antiviral efficacy estimates with previous studies.

Studies	Treatment	HBeAg	Number of patients	Study period(weeks)	V_t 1/2_ (hours)	I_t 1/2_(days)	Antiviral efficacy (%)
Nowak et al. (7)	LAM	+	23	4	24.0	10–100	87–99
Tsiang et al. (13)	ADV	+	10	12	26.4	11–30	99
Lau et al. (8)	LAM/LAM+FCV	+	21	12	48.3	43	99/94
Lewin et al. (10)	LAM/LAM+FCV	+	15	12	28.5	2.4– >120	95/99
Wolters et al. (12)	LAM	+	21	4	13.0	<0–331	92/96
Wolters et al. (11)	LAM/LAM+FCV/LAM+GCV	+/−	12	4	12.7	3–26	93/95/86
Wolters et al. (14)	ETV	+/−	10	4	16.0	5.2–31.8	87–98
Sypsa et al. (15)	PEG-IFN/PEG-IFN+ LAM/LAM	−	44	4	12.7	2.7–75	83/93/96
Our study	CLV	+	50	24	24.9	7.4	97

Abbreviations: V_t 1/2_, half-life of free virus; I_t 1/2_, half-life of infected hepatocytes; LAM, lamivudine; FCV, famciclovir; ADV, adefovir; ETV, entecavir; GCV, ganciclovir; PEG-IFN, pegylated interferon; CLV, clevudine.

A median of 7.39 days as an estimated half-life of the infected cells was comparable to estimates from previous studies (Table 5). This finding can be simply translated into a 6.8% daily loss in the infected cell population. Given that 5–40% of all hepatocytes in CHB patients are productively infected, although there is no agreement on the number of infected cells, this calculation means that between 0.3–2.7% of all hepatocytes are hypothetically killed daily and they must be replenished to maintain the liver mass in a stable state. It has been shown that hepatocyte turnover rate would be much faster if the number of infected cells is close to 100%. [34], [35] However, this hypothetical condition was deemed less likely in our patients because continuous daily turnover at such rate should have caused persistently elevated ALT, and this was not observed in our patients (data not shown).

Whether the HBV infection is cleared or persistent is determined by a complex interaction between the virus and the host immune system. [36] Chisari et al. have demonstrated that more than 90% of the viral DNA can be eliminated from the liver by noncytolytic processes; the viral cccDNA was also found susceptible to noncytolytic control in a study of acute HBV infection in chimpanzees. [27] Unlike acute infection, chronic HBV infection is thought to be maintained by the cccDNA; the limiting factor in eliminating infection is presumably the clearance of the cccDNA reservoirs from the infected cells. [37] Two immune mechanisms have been proposed to mediate the cccDNA clearance in CHB: one is a cytolytic mechanism by which infected cells are eliminated and replaced by cells from an uninfected lineage [38], [39]; the other involves a noncytolytic, cytokine-induced “curing” of infected cells. [24], [27] Thus, we made an attempt to incorporate the noncytolytic immune process into our modeling. Unlike the cytolytic activity which can be represented by serum ALT levels, quantitative information on the noncytolytic activity during antiviral treatment can hardly be achieved without extensive serial liver biopsies and quantification of intrahepatic cccDNA; these procedures are not easily performed in either clinical studies or practice. On the other hand, serial serum HBsAg titers were recently reported to indirectly reflect the intracellular cccDNA concentrations. [40] Quantitative HBsAg analyses of baseline value or on-treatment decline were also reported to be useful in predicting response to antiviral treatment, suggesting linkage between HBsAg and immune activity. [41], [42] In this study, we obtained data on serial HBsAg titers matched with HBV DNA in each patient and therefore referred to HBsAg titers to be informed of the behavior of immune activities. In addition, the concept of negative feedback was also introduced in the equation of immune effectors “E” as described. [21] Thus, we solved the model equations using the literature values and the assumptions as described above; the viral decay pattern was triphasic in these patients (Fig. 1). The HBV DNA decline flattens in a significant portion of the patients after 4–5 weeks of antiviral treatment; this effect might be caused by reduction of the clearance rate of the infected cells over time [33]. This phenomenon was described as “negative feedback” by Colombatto et al.; the progressive reduction of the infected cell clearance rate which is proportionate to the decline of the infected cells during treatment. [21] We designed our model equations with keeping this concept in mind, in order to introduce the activity of immune effectors. [28], [29] Our parameter estimation was successful in the study subjects when serial HBsAg titers were used as a guide toward the behavior of non-cytolytic immune activities.

In Table 4 and Figure 2, the correlation analysis revealed a significant correlation between the baseline HBV DNA and the half-life of the infected cells. The baseline HBV DNA also showed an association with the baseline HBsAg titer, which is known to reflect the total amount of cccDNA in the liver (Fig. 2-A). [40] Given that the higher HBsAg titer and the HBV DNA suggest the presence of a substantial cccDNA content in the liver, the half-life of the infected cells might be prolonged during treatment (Fig. 2-B). High baseline HBV DNA was associated with steeper slopes of phase 1 and 2, which in turn showed association with enhanced on-treatment decrease in HBsAg titer (Fig. 2-C; Fig. 2-D). This finding could be interpreted as delayed negative feedback and consequently enhanced HBsAg reduction due to high initial viral load. Parameter “α” of the model equation, which represented the clearance rate of the infected cells induced by cytolytic immune activity, revealed correlation with antiviral efficacy. (Fig. 2-E), Parameter “α” was inversely correlated with the baseline HBV DNA (Fig. 2-F), which is in agreement with previous studies where antiviral therapy showed better results in case of lower HBV DNA.[43]–[45] However, the correlations of “α” were not robust, which might be related to the following possibilities: i) need for further refinement of the model equations; ii) outweighing relevance of noncytolytic mechanism during antiviral therapy rather than cytolytic immune activity. [40], [46].

There are some limitations in this study. First, given that viral dynamics vary according to various genotypes of the HCV, the effect of genotypes on the HBV dynamics needs to be elucidated, [47] although virtually all patients with CHB in Korea are known to be infected with HBV of genotype C. [48] Second, this study included only HBeAg-positive subjects. For these reasons, generalization of our methodology needs further verification in patient populations with HBeAg-negative CHB. Third, and more importantly, we only included “responsive” patients with continuously decreasing HBV DNA and on-treatment decline of HBsAg titers. These inclusion criteria prevent application of our modeling method to patients showing a complex decay pattern or to those without HBsAg decline.

In conclusion, the present study demonstrated that the mathematical modeling of the HBV dynamics during 24 weeks of antiviral therapy was successful with adding immune effector as a new compartment in the model equations. Some of the estimated parameters demonstrated significant correlation with observed biologic phenomena, e.g., on-treatment viral decline and negative feedback of immune activity. The resultant triphasic dynamics seems more appropriate to explain long-term viral dynamics compared to the traditional biphasic model. Our methodology of modeling needs to be validated in clinical studies using other NUCs available for patients with CHB, to examine the possibility of offering insights for appropriate treatment endpoint or optimal treatment duration.
